# Understanding adolescent consumption patterns and attitudes towards the “puff” on the path to a smoke-free generation: a 2022 French perspective

**DOI:** 10.3389/fpubh.2024.1411099

**Published:** 2024-09-20

**Authors:** Marin Cottin, Marion Catellin, Elen De Guiran, Katiuska Miliani, Loïc Josseran, Sylvain Gautier

**Affiliations:** ^1^Department of Epidemiology and Public Health, Raymond Poincaré Hospital, Garches, France; ^2^INSERM UMR1018, Primary Care and Prevention Team, Paris-Saclay University and Versailles Saint-Quentin-en-Yvelines University, Villejuif, France; ^3^Academic Department of Public Health, Prevention, Observation, Territories, UFR Simone Veil, Health, Versailles Saint-Quentin-en-Yvelines University, Montigny-le-Bretonneux, France; ^4^ACT—French Alliance Against Tobacco, Paris, France

**Keywords:** tobacco, public health, electronic cigarette, smoking prevention, adolescents

## Abstract

**Purpose:**

Tobacco use remains the leading preventable cause of death in France, with 75,000 deaths each year. France aims to reduce smoking and achieve a smoke-free generation by 2032. However, recent tobacco industry innovations which mainly target young people, could undermine this goal. The main objective of this study is to assess the knowledge and consumption patterns of the “puff” among French adolescents in 2022.

**Methods:**

A cross-sectional study using a structured online survey on a representative sample of 400 adolescents aged 13 to 16 years was conducted from July 4th to 20th, 2022.

**Results:**

Around 66% of adolescents reported having heard of the puff”, and one in ten having tried it. Slightly fewer of them have tried cigarettes; 89.6% of experimenters reported that it allowed them to explore unique flavors, 81.9% found it fun to play with the puff-cloud, and 94.5% of regular consumers considered it a stylish or cool product. 76% of adolescents believe that the puff is dangerous to their health, 71.6% describe it as a polluting device, and 62.8% think it’s a gadget.

**Conclusion:**

The “puff” is widely known by French adolescents and more commonly used than cigarettes, due, in part, to marketing specifically designed to target youth. That is why it could represent a threat to the smoke-free generation objectives. Public health policy could be informed by the ecological awareness of adolescents as a new lever of counter-influence to prevent this kind of consumption, as did ACT with the #stopcigarettespollution prevention campaign.

## Highlights

- **What is already known on this topic?**

The “Puff Bar,” a disposable electronic cigarette introduced in 2019, has quickly become popular among adolescents due to its colorful packaging, variety of flavors, affordability, and ease of concealment and use.

- **What this study adds?**

This study provides initial insights into French adolescents’ perceptions of the “puff.” It found widespread awareness and significant experimentation, with varied views on its cost, environmental impact, and style, alongside peer and social media influences and recognized addiction risks.

- **How might this study affect research, practice or policy?**

The study highlights the challenge for public health authorities to respond to market innovations like the “puff.” Leveraging ecological concerns, to which the youth are sensitive, could be key to achieving a smoke-free generation.

## Introduction

The battle against tobacco use has evolved into a global priority in public health (1). Across the world, governments and health organizations have united in their efforts to combat the devastating consequences of tobacco addiction and use of tobacco products (2).

In France, while historical milestones such as the Veil Law of 1976 and the Evin Law of 1991 have undoubtedly played significant roles in tobacco control, the quest for effective strategies continues (3). In recent years, there has been an escalation of anti-tobacco measures, including steep increases in tobacco prices, greater accessibility to nicotine replacement therapies, and innovative social marketing campaigns, exemplified by initiatives like “Mois sans Tabac” (Tobacco-Free Month) and graphic health warnings on cigarette packaging.

The introduction of plain packaging regulations and the ban on flavored additives, particularly those appealing to the young palate, have been noteworthy milestones in curtailing the industry’s influence. Furthermore, the creation of a dedicated fund for tobacco prevention underscores the commitment of the French government to address tobacco addiction. Within this framework, the National Tobacco Control Program (*Programme National de Lutte contre le Tabac*, PNLT) has laid out a comprehensive strategy with ambitious goals. By 2020, the aim was to reduce the prevalence of daily smokers among 18–75 year-olds to below 24%. Looking ahead to 2022, the target extends to a daily smoking rate of less than 22% among 18–75 year-olds and a rate below 20% among those under 17 years of age.

Despite an overall population prevalence of 25%, this program has also introduced the goal of achieving a “smoke-free generation” (“*la première generation sans tabac*”) by 2032, which, under this term, means reducing the prevalence of tobacco use among young people to less than 10% (4). However, this mission is not without its challenges, as the tobacco industry has demonstrated a remarkable capacity to adapt and circumvent the measures aimed at curtailing its influence.

One of the industry’s most persistent strategies has been to target the youth demographic, viewing it as the linchpin in subverting the public health vision of a smoke-free generation. For decades, the tobacco industry has spared no effort in enticing young people by employing tactics that have ranged from seductive advertising to the development of innovative nicotine delivery systems (5). The ultimate objective of the tobacco industry is to cultivate dependence and ensure the perpetuation of a customer base that sustains the tobacco industry for generations to come.

In this ongoing tug-of-war between public health and corporate interests, one recent entrant has emerged as a formidable challenge—the “puff.” Born in the United States in 2019, under the trade name “Puff Bar,” this disposable electronic cigarette has rapidly gained traction among adolescents over the past 2 years under the now generic name of the “puff” (6). Its appeal is multifaceted, aligning perfectly with the desires and preferences of young people. Featuring colorful packaging and a tantalizing assortment of flavors, the “puff” is not only affordable but also readily accessible. These disposable electronic cigarettes are also more compact and concealable than other types of electronic cigarettes (tank-style, pod mod, etc.), making it easier for them to be used without detection by parents supervision or other forms of authority (7). Furthermore, they are inexpensive and simpler to use for young consumers (8).

In France, the diffusion of the “puff” is probably similar as the other countries but there is a lack of epidemiological evidence about this phenomenon. Despite recent action taken by the French government to ban sales of the “puff” (9), a pressing need persists to comprehensively understand the consumption habits of adolescents and their perceptions of this enticing product. The advent and rapid proliferation of the “puff” among adolescents pose a new and pressing challenge. Just as it has been suggested in the context of electronic cigarettes, the use of such products by younger individuals raises the question of initiation into smoking of combusted products (gateway phenomenon) (10).

Thus, the objective of this study is to assess the knowledge and consumption patterns of the “puff” among French adolescents in the year 2022. By delving into the intricate facets of this phenomenon, we aim to provide valuable insights that will inform policy, guide public health interventions, and contribute to the broader dialogue surrounding adolescent tobacco use.

## Methods

### Study design and data collection

A cross-sectional study was conducted using a structured online survey, conducted from July 4th to July 20th, 2022 by a professional polling institute (the BVA group). The survey was designed to gather data from a nationally representative sample of adolescents aged 13 to 16 years, with recruitment facilitated through parental involvement. The sampling methodology followed the principle of quota sampling, with careful consideration of the following variables: gender and age of the adolescent, the occupation of the household’s reference person, geographic region, and urbanization level.

In order to determine the number n of adolescents to survey, and thus the sample size, we use the following formula: n=z2×p1−pe21+z2×p1−pe2×N , with z = 1.96 corresponding to a desired confidence level of 95% in the estimates, e = 0.05 corresponding to a 5% margin of error in the estimates, and N representing the size of the reference population (here, 3,307,998). The optimal sample size is calculated with p = 0.5. The estimated sample size is 385.

### Sample selection and representativeness

The initial recruitment of participants was facilitated through their parents or guardians. The recruitment process adhered to a multistage stratified sampling approach, ensuring that the sample composition aligned with the demographic distribution of adolescents in France. The primary stratification criteria included gender, age group, region, and urbanization level. In doing so, we ensured that the survey sample accurately reflected the population distribution of adolescents in France.

To guarantee representativeness, data weighting was applied to adjust for any discrepancies between the survey sample and the population at large. This process involved assigning appropriate weights to individual survey responses based on the demographic characteristics of the reference population. These weighted responses were then analyzed, enabling us to present the findings as representative of the broader adolescent population in France.

### Survey instrument

The survey instrument was designed by the research team to capture a comprehensive range of the following domains:

- Tobacco and “puff” consumption: participants were queried about their history of tobacco and “puff” usage, including the frequency, duration, and reasons behind their consumption. Detailed information regarding the types of tobacco products and flavors of “puff” used was also collected;
- Knowledge and perceptions: adolescents’ awareness and perceptions of the “puff,” its risks, and its appeal were assessed. The survey aimed to understand how marketing and packaging influenced their perceptions and decisions regarding usage;
- Accessibility: the survey probed the ease with which adolescents could access tobacco and “puff” products, encompassing the channels through which they obtained them.

### Ethical considerations

The study adhered to ethical standards, with all data collected anonymously to safeguard the privacy of participants. Informed consent was sought from parents or guardians before survey participation, and assent was obtained from the adolescents. It was declared to the CNIL (*Commission Nationale de l’Informatique et des Libertés*) under the number 2231485, ensuring that the study adhered to relevant legal and ethical guidelines, thereby safeguarding the privacy and rights of study participants. The study was approved by the Foch IRB: IRB00012437 (Approval Number: 24-09-01).

### Data analysis

Data collected were analyzed using *R*, version 4.0. Descriptive statistics, such as frequencies, means, and standard deviations, were employed to provide an overview of the data. Inferential statistics, including chi-square tests and logistic regression, were utilized to explore associations and relationships within the dataset. The table data include raw counts and adjusted percentage proportions.

## Results

### Participant characteristics

Out of the 400 respondents, 51% were boys (*n* = 204), and 49% were girls (*n* = 196) (Table 1). Nearly half of the sample consisted of 13–14 year-olds (*n* = 199, 49.8%). Approximately 20% of the respondents lived in the Paris region (*n* = 64, 16%), and the vast majority were in middle school. Adolescents from more advantaged households (parents’ socio-economic category considered as privileged) constituted the majority at 53.7% (*n* = 215). Many adolescents reported that both of their parents did not smoke (*n* = 215, 53.8%).

**Table 1:** Characteristics of the sample (*N* = 400).

	Total^a^ *N* = 400 (100%)	Boys *n* = 204 (51%)	Girls *n* = 196 (49%)
**Age category**			
[13–14] y.o.	199 (49.8)	107 (52.0)	93 (47.5)
[15–16] y.o.	201 (50.2)	98 (48.0)	103 (52.5)
**Live place**			
Rural or small city	168 (42.0)	91 (44.6)	76 (39.2)
Great or medium sized city	168 (42.0)	86 (41.8)	82 (42.2)
Paris area	64 (16.0)	28 (13.6)	36 (18.6)
**School stream** ^b^			
General	368 (92.1)	189 (92.2)	179 (92.0)
Technological	11 (2.7)	5 (2.6)	6 (2.9)
Professional/agricultural	21 (5.2)	11 (5.2)	10 (5.2)
**Parents’ socio-economic category**			
Privileged	215 (53.7)	111 (54.3)	103 (53.0)
Disadvantaged	174 (43.4)	90 (44.0)	83 (42.7)
Unemployed	12 (3.0)	3 (1.7)	8 (4.3)
**Parents tobacco status**			
Smoker	185 (46.2)	96 (46.9)	89 (45.4)
Non-smoker	215 (53.8)	109 (53.1)	107 (54.6)

Puff ACT study, France, July 2022.

a In this table, the raw counts are followed by adjusted proportions (expressed as percentages).

b In French high school, students are divided into diverse programs. The general program prepares students for university and preparatory classes, the technological program prepares students for short post-graduate studies, and the professional and agricultural program provides cooperative education for a quick entry into the professional world.

### Awareness and knowledge of the “puff”

**Figure 1:**
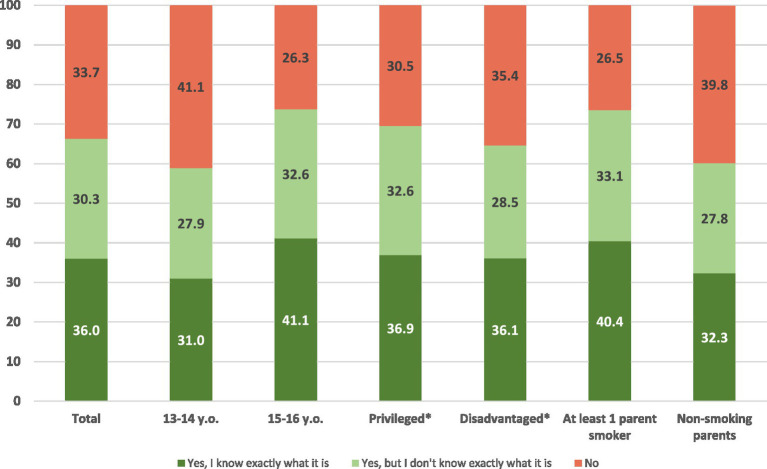
Knowledge of the “puff.” ACT study, France, July 2022. *Refer to the parents’ socio-economic category.

Overall, nearly 66% of adolescents reported having heard of the “puff” (Figure 1). Of these, 36% indicated that they knew precisely what it was, while 30% were aware of it without precise knowledge. Among 15–16 year-olds, 74% had heard of the “puff.” Adolescents from more advantaged households were also more likely to be aware, with 72% reporting knowledge of it. When at least one parent smoked, adolescents reported awareness at a rate of 74%. Conversely, 59% of 13–14 years-olds and 61% of adolescents from less advantaged households reported awareness of the “puff.”

### Usage patterns

More than one in ten adolescents reported having tried the “puff”: 12.9% reported having tried it (Table 2). In our sample, young girls appear to experiment more with the “puff” than boys (14.7% versus 11.2%), although this difference is not statistically significant. When at least one parent smoked, 20% of adolescents reported having tried it, and this rate increased to 29% when both parents smoked. Additionally, 5.2% of adolescents reported occasional “puff” use, with a disparity observed between girls (5.5%) and boys (4.9%) without it being significant. Experimentation with tobacco and e-cigarettes followed similar patterns, with 14.9% of adolescents reporting ever having tried or used e-cigarettes, and 12.5% reporting ever having tried or used traditional cigarettes or rolling tobacco.

**Table 2:** Consumptions of tobacco products.

	Total^a^ *N* = 400 (100%)	Boys *n* = 204 (51%)	Girls *n* = 196 (49%)
**Cigarettes**			
Experimentation	50 (12.5)	26 (12.9)	24 (12.2)
Current consumption	18 (4.5)	10 (4.9)	8 (4.1)
**Heated tobacco**			
Experimentation	19 (4.8)	9 (4.3)	10 (5.3)
Current consumption	4 (1.1)	2 (1.2)	2 (0.9)
**Snus**			
Experimentation	7 (1.7)	5 (2.4)	2 (1.1)
Current consumption	3 (0.8)	3 (1.5)	0 (0)
**Shisha**			
Experimentation	34 (8.5)	16 (7.7)	18 (9.4)
Current consumption	4 (0.9)	1 (0.3)	3 (1.6)
**E-cigarette**			
Experimentation	60 (14.9)	31 (15.3)	28 (14.5)
Current consumption	19 (4.8)	10 (4.8)	9 (4.7)
**Puff**			
Experimentation	52 (12.9)	23 (11.2)	29 (14.7)
Current consumption	21 (5.2)	10 (4.9)	11 (5.5)

Puff ACT study, France, July 2022.

a In this table, the raw counts are followed by adjusted proportions (expressed as percentages).

Among users of the “puff,” 28% had transitioned to consuming it. Of these, 17% subsequently started using other tobacco products, notably cigarettes or rolling tobacco, while 11% continued to use the “puff.” Overall, 45% of adolescents who reported using tobacco products indicated that they had initiated their consumption with cigarettes or rolling tobacco. This percentage increased to 57% when at least one parent smoked.

### Perception of the “puff”

The majority of respondents regarded the “puff” as “a gadget” (81.5%) i.e. just a gimmick, “polluting” (71.6%), or “too expensive” (62.8%) (Table 3). Among experimenters, 89.6% reported that it allowed them to explore unique flavors, and 81.9% found it fun to play with the “puff” cloud. Among regular “puff” consumers, 94.5% considered it as a stylish or cool product. When at least one parent smoked, 68% (compared to 61%) of adolescents believed that it allowed them to discover unique flavors, and 75% did so when both parents smoked. The favorite flavors among users are strawberry (28.7%) and mint (10.4%), followed by tobacco (9.1%).

**Table 3:** Perception of the puff.

	Total *N* = 262	Experimenters *n* = 51*	Consumers *n* = 21
Stylish/Cool	123 (47.1)	42 (80.6)	20 (94.5)
Funny	135 (52.1)	42 (81.9)	18 (86.4)
Allow to discover original flavor	160 (61.0)	46 (89.6)	19 (90.5)
Gadget	213 (81.5)	41 (78.9)	16 (73.2)
Too expensive	164 (62.8)	31 (61.1)	11 (51.4)
Polluting	187 (71.6)	26 (52.8)	5 (24.6)

Puff ACT study, France, July 2022. *1 missing data. In this table, the raw counts are followed by adjusted proportions (expressed as percentages).

Up to 30% of adolescents expressed a desire to use the “puff” when they saw others using it on social media. This proportion increased to 40% when at least one parent smoked and 54% when both parents were smokers. In contrast, the temptation to use e-cigarettes (14%) (rechargeable/reusable electronic devices, excluding puffs), and hookahs (9%) was less pronounced when influenced by social media.

Adolescents demonstrated awareness of addiction risks, with 84% stating that e-cigarettes “can be addictive” and 82% making the same assertion about the “puff.” This awareness extended to 86% when adolescents had never used the “puff.” Moreover, 76% believed that the “puff” was dangerous to their health, with 48% categorizing it as “rather dangerous” rather than “very dangerous” (28%).

### Accessibility

One-quarter of adolescents reported easy access to the “puff.” This was facilitated for adolescents from more advantaged families (35% reported easy access) and those with both parents who smoked (42% reported easy access). Similar patterns were observed for cigarettes and e-cigarettes. Notably, 10% of adolescents aged 13 to 16 had purchased a “puff,” a rate significantly higher among those who were aware of it (40%). The school setting was the primary place of product discovery (for 50% of the respondents) and, also the main location where puff was used (49.7% of the respondents).

## Discussion

Our study represents the first study conducted in France to date on the usage and perception of the puff by adolescents. It revealed several notable findings: approximately two-thirds of respondents were aware of the “puff,” with a significant proportion having tried it (12.9%), often in conjunction with experimentation of other tobacco and nicotine products. Despite this high level of experimentation, perceptions of the “puff” ranged from it being seen as too expensive and polluting to a gadget or a stylish product, depending on the perspective of users and non-users. It is also significant to note that many adolescents reported temptations influenced by peers and social media, while a substantial proportion recognized the potential risks of addiction associated with such products.

These results are similar to those that have been highlighted in previous studies conducted in other countries, notably in the United Kingdom (11). Some of these studies, conducted from a qualitative perspective, have been able to explore the significant marketing influence of these disposable electronic cigarettes on adolescents (7). The perception that puffs are too expensive could be due to a lack of awareness of the actual selling price or a perception of higher cost relative to the number of uses permitted by a puff compared to a pack of cigarettes.

Although the differences between boys and girls were not significant in our study, it appears that young girls may be more inclined to consume the “puff” on a daily basis. This is consistent with literature on the subject, as evidenced by a 2022 study conducted in the UK which showed that women were spontaneously more attracted to this product (11). Furthermore, our results reveal a higher proportion of adolescent consumers among the more privileged socio-economic categories, even though this demographic is traditionally less inclined towards tobacco product consumption (12).

This study has both strengths and limitations. First, point estimates should also be considered with the 5% margin of error in mind. Secondly, the sample size is relatively small, and the proportion of children from socio-economically privileged families is overrepresented compared to the general population. This can be explained by the individual and family resources required to complete the online survey (material resources such as computer and Internet connection, as well as cognitive resources, such as mastery of French), or by a social desirability bias. However, we have applied data weighting to our sample to ensure its representativeness across key demographic variables (including parent’s socio-economic status), making it a valuable resource for understanding the knowledge and consumption patterns of the “puff” among French adolescents. However, the study’s cross-sectional nature necessarily limits our ability to trace the trajectory of consumption patterns over time. In the same way, the limitation of requiring internet access for answering the questionnaire is addressed by employing a quota sampling method, which, while not entirely eliminating selection bias, ensures that the sample remains balanced and representative of the broader French population. It was not possible, given the chosen sampling model and the ethical considerations justifying the need to survey the parents before administering the questionnaire to the adolescents, to include participants from families with no Internet access or with low digital literacy. It is important to note that this study appears to be the first of its kind in France, shedding light on a previously unexplored area in population-based research.

Regarding a primary concern associated with the use of the “puff,” which is the potential gateway phenomenon to smoking it may represent, our study does not formally allow us to draw conclusions due to the inherent methodological limitations of this cross-sectional type of study (13). However, it is worth noting that in our study, 28% of adolescent puff consumers reported initiating their combusted tobacco consumption after starting to use the “puff.”

The findings of this study underscore the pressing need for robust regulation and control measures in the realm of tobacco products and adapting in real-time to new innovations of the tobacco industry including novel devices like the “puff.” As the tobacco industry continues to adapt and diversify its offerings, it becomes increasingly clear that these innovations are not only intended to meet current demands but also to shape the preferences and behaviors of young people (14). The “puff” with its colorful packaging, diverse flavors, and affordability, exemplifies the industry’s agility in targeting young consumers (15). Furthermore, as shown in our study, the high proportion of adolescents who have already used the “puff” and the ease of access to this product raise questions about compliance with the current regulations regarding the sale of tobacco products, which in France has been restricted to individuals over 18 years old for a long time. This may also reflect a need for more aggressive enforcement of these policies. The decision by the French government to ban the sale of the “puff” reflects a proactive step in addressing the proliferation of such products among adolescents (16). More broadly, there is a need to strengthen the enforcement, particularly among tobacconists, of the ban on the sale of tobacco to minors. However, to reduce increases in tobacco product use, it is essential to remain vigilant in the face of evolving tobacco industry’ marketing strategies and to continuously adapt regulations to encompass the ever-expanding array of tobacco and nicotine delivery systems. Beyond immediate control and prohibition measures, it also seems necessary to support adolescents in adopting healthy behaviors and to develop health-promoting environments that reduce the risk of starting to smoke.

One particularly—promising finding—is the environmental awareness demonstrated by adolescents. A significant proportion recognized the environmental concerns associated with the “puff,” which is a disposable product made of plastic and lithium batteries. This awareness presents an opportunity for public health authorities to emphasize the environmental impact of such products as part of their denormalization communication strategy (17). By linking the use of disposable e-cigarettes to environmental issues, health organizations could resonate with the ecological aspirations held by many young people (18). Addressing the environmental consequences of such products may serve as a powerful deterrent and encourage adolescents to make more health-conscious choices.

The study’s broader implications raise important questions about how public health can effectively respond to rapidly evolving market innovations, especially those aided by the influence of social media and the ease with which products circulate globally (19). The “puff” phenomenon demonstrates the challenges posed by the capacity of the tobacco industry to create products that can quickly gain popularity, particularly among young and vulnerable populations. Public health strategies must adapt and anticipate these innovations to protect the health of individuals and communities. This includes continually monitoring emerging products, conducting research on it and implementing evidence-based interventions. Equally important is the need for international collaboration to address products that can easily cross borders.

## Data Availability

The raw data supporting the conclusions of this article will be made available by the authors, without undue reservation.
